# Butane-1,4-diyl bis­(pyridine-3-carboxyl­ate)

**DOI:** 10.1107/S160053681202260X

**Published:** 2012-06-13

**Authors:** Javier Vallejos, Iván Brito, Alejandro Cárdenas, Michael Bolte

**Affiliations:** a Departamento de Química, Facultad de Ciencias Básicas, Universidad de Antofagasta, Casilla 170, Antofagasta, Chile; b Departamento de Física, Facultad de Ciencias Básicas, Universidad de Antofagasta, Casilla 170, Antofagasta, Chile; c Institut für Anorganische Chemie der Goethe-Universität Frankfurt, Max-von-Laue-Strasse 7, D-60438 Frankfurt am Main, Germany

## Abstract

Mol­ecules of the title compound (alternative name: butane-1,4-diyl dinicotinate), C_16_H_16_N_2_O_4_, lie on a inversion centre, located at the mid-point of the central C—C bond of the aliphatic chain, giving one half-mol­ecule per asymmetric unit. The butane chain adopts an all-*trans* conformation. The dihedral angle between the mean plane of the butane-3-carboxyl­ate group [for the non-H atoms, maximum deviation = 0.0871 (15) Å] and the pyridine ring is 10.83 (7)°. In the crystal, mol­ecules lie in planes parallel to (122). The structure features weak π–π inter­actions with a centroid–centroid distance of 3.9281 (11) Å.

## Related literature

For the crystal structures of compounds with related ligands, see: Brito *et al.* (2010**a* ▶,*b* ▶,c* ▶, 2011 ▶).
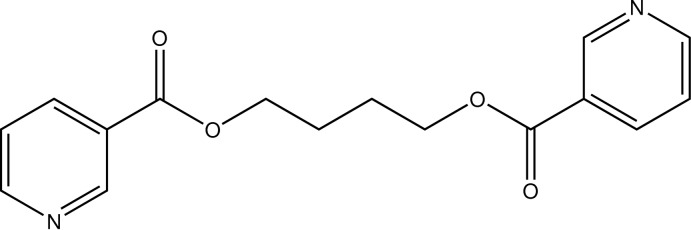


## Experimental

### 

#### Crystal data

C_16_H_16_N_2_O_4_
*M* *_r_* = 300.31
Triclinic, 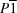

*a* = 6.7186 (10) Å
*b* = 7.6942 (12) Å
*c* = 8.2462 (13) Å
α = 65.290 (11)°
β = 75.499 (12)°
γ = 68.207 (11)°
*V* = 357.28 (10) Å^3^
*Z* = 1
Mo *K*α radiation
μ = 0.10 mm^−1^
*T* = 173 K
0.29 × 0.25 × 0.18 mm

#### Data collection

Stoe IPDS II two-circle diffractometer
Absorption correction: multi-scan (*X-AREA* and *X-RED32*; Stoe & Cie, 2001 ▶) *T*_min_ = 0.971, *T*_max_ = 0.982
6958 measured reflections
1347 independent reflections
1171 reflections with *I* > 2σ(*I*)
*R*_int_ = 0.035

#### Refinement

*R*[*F* ^2^ > 2σ(*F* ^2^)] = 0.038
*wR*(*F* ^2^) = 0.090
*S* = 1.13
1347 reflections
100 parameters
H-atom parameters constrained
Δρ_max_ = 0.19 e Å^−3^
Δρ_min_ = −0.14 e Å^−3^

### 

Data collection: *X-AREA* (Stoe & Cie, 2001 ▶); cell refinement: *X-AREA*; data reduction: *X-RED32* (Stoe & Cie, 2001 ▶); program(s) used to solve structure: *SHELXS97* (Sheldrick, 2008 ▶); program(s) used to refine structure: *SHELXL97* (Sheldrick, 2008 ▶); molecular graphics: *PLATON* (Spek, 2009 ▶); software used to prepare material for publication: *SHELXL97*.

## Supplementary Material

Crystal structure: contains datablock(s) I, global. DOI: 10.1107/S160053681202260X/su2433sup1.cif

Structure factors: contains datablock(s) I. DOI: 10.1107/S160053681202260X/su2433Isup2.hkl

Supplementary material file. DOI: 10.1107/S160053681202260X/su2433Isup3.cml

Additional supplementary materials:  crystallographic information; 3D view; checkCIF report

## Figures and Tables

**Fig. 1.**
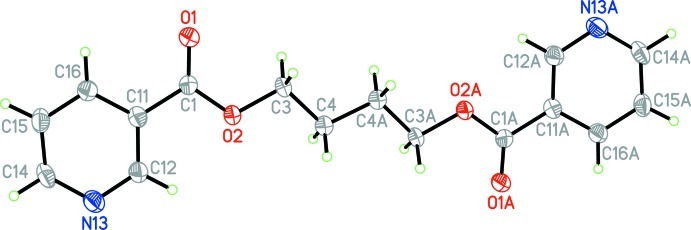
A view of the molecular structure of the title molecule, with the atom-numbering. Displacement ellipsoids are drawn at the 50% probability level [symmetry code: (A) = -x+2, -y+2, -z].

**Fig. 2.**
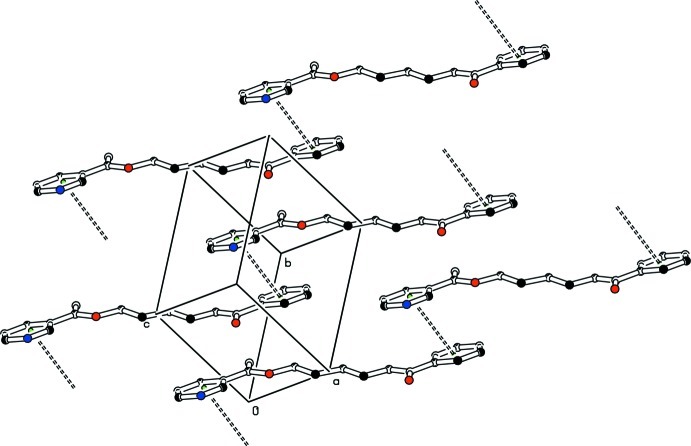
A partial view of the crystal packing of the title compound, showing the weak π–π stacking interactions [Cg = centroid of ring N13,C11,C12,C14-C16; symmetry code: (i) = -x+1, -y+1, -z+1; H atoms have been omitted for clarity].

## supplementary crystallographic information

### Comment

This paper forms part of our continuing study of the synthesis and structural characterization of coordination polymers (Brito *et al.*, 2010**a*,*b*,*c*;* 2011). We are particularly interested in the utility of the title compound as a flexible ligand and for its binding modes, in order to synthesis different coordination polymer topologies. Molecules of the title compound, (Fig. 1), lie on an inversion centre, which passes through middle point of the C4—C4A bond of the aliphatic chain, giving one half-molecule per asymmetric unit. The butane chain adopts an all *trans* conformation. The dihedral angle between the mean plane of the butane-3-carboxylate moiety [non-H atoms; max. deviation for atoms C3/C3A = 0.0871 (15) Å] and the pyridine ring is 10.83 (7)°.

The crystal structure is stabilized by weak π–π interactions with centroid-centroid distances of 3.9281 (11) Å. The molecules lie in planes parallel to (122) [Fig. 2].

### Experimental

Isonicotinoyl chloride hydrochloride (354 mg, 2 mmol) was taken in a 50 ml round bottom schlenk flask and fitted with a reflux condenser. Dichloromethane 25 ml, 1,4-Butanediol (0.10 ml, 1.0 mmol) and 1 ml of triethylamine were added. The reaction mixture was heated at 323 K for 3 h. After, the mixture was washed with saturated aqueous sodium bicarbonate solution (50 ml), the organic layer was dried over anhydrous sodium sulfate and filtered. The solvent was evaporated using vacuum and the white product was purified by recrystallization with acetonitrile (Yield: 87%). Single crystals suitable for X-ray diffraction were prepared by slow evaporation of a solution of the title compound in dichloromethane at room temperature.

Spectroscopic details are given in the archived CIF.

### Refinement

All H-atoms were positioned geometrically with C—H = 0.95 or 0.99 Å and refined using a riding model with *U*_iso_(H) = 1.2*U*_eq_(C).

### Crystal data

**Table:**

C_16_H_16_N_2_O_4_	*Z* = 1
*M**_r_* = 300.31	*F*(000) = 158
Triclinic, *P*1	*D*_x_ = 1.396 Mg m^−^^3^
Hall symbol: -P 1	Mo *K*α radiation, λ = 0.71073 Å
*a* = 6.7186 (10) Å	Cell parameters from 9708 reflections
*b* = 7.6942 (12) Å	θ = 3.3–26.0°
*c* = 8.2462 (13) Å	µ = 0.10 mm^−^^1^
α = 65.290 (11)°	*T* = 173 K
β = 75.499 (12)°	Block, colourless
γ = 68.207 (11)°	0.29 × 0.25 × 0.18 mm
*V* = 357.28 (10) Å^3^	

### Data collection

**Table:**

Stoe IPDS II two-circle diffractometer	1347 independent reflections
Radiation source: Genix 3D IµS microfocus X-ray source	1171 reflections with *I* > 2σ(*I*)
Genix 3D multilayer optics monochromator	*R*_int_ = 0.035
ω scans	θ_max_ = 25.6°, θ_min_ = 3.3°
Absorption correction: multi-scan (*X-AREA* and *X-RED32*; Stoe & Cie, 2001)	*h* = −7→8
*T*_min_ = 0.971, *T*_max_ = 0.982	*k* = −9→9
6958 measured reflections	*l* = −10→10

### Refinement

**Table:**

Refinement on *F*^2^	Primary atom site location: structure-invariant direct methods
Least-squares matrix: full	Secondary atom site location: difference Fourier map
*R*[*F*^2^ > 2σ(*F*^2^)] = 0.038	Hydrogen site location: inferred from neighbouring sites
*wR*(*F*^2^) = 0.090	H-atom parameters constrained
*S* = 1.13	*w* = 1/[σ^2^(*F*_o_^2^) + (0.0318*P*)^2^ + 0.1244*P*] where *P* = (*F*_o_^2^ + 2*F*_c_^2^)/3
1347 reflections	(Δ/σ)_max_ < 0.001
100 parameters	Δρ_max_ = 0.19 e Å^−^^3^
0 restraints	Δρ_min_ = −0.14 e Å^−^^3^

### Special details

**Table:**

Experimental. Spectroscopic details for the title compound:IR (KBr): 3087 (w), 1713 (s), 1589 (m), 1473 (w), 1294 (s), 1195 (s), 741(m), cm^-1^.
Geometry. All e.s.d.'s (except the e.s.d. in the dihedral angle between two l.s. planes) are estimated using the full covariance matrix. The cell e.s.d.'s are taken into account individually in the estimation of e.s.d.'s in distances, angles and torsion angles; correlations between e.s.d.'s in cell parameters are only used when they are defined by crystal symmetry. An approximate (isotropic) treatment of cell e.s.d.'s is used for estimating e.s.d.'s involving l.s. planes.
Refinement. Refinement of *F*^2^ against ALL reflections. The weighted *R*-factor *wR* and goodness of fit *S* are based on *F*^2^, conventional *R*-factors *R* are based on *F*, with *F* set to zero for negative *F*^2^. The threshold expression of *F*^2^ > σ(*F*^2^) is used only for calculating *R*-factors(gt) *etc*. and is not relevant to the choice of reflections for refinement. *R*-factors based on *F*^2^ are statistically about twice as large as those based on *F*, and *R*- factors based on ALL data will be even larger.

### Fractional atomic coordinates and isotropic or equivalent isotropic displacement parameters (Å^2^)

**Table:**

	*x*	*y*	*z*	*U*_iso_*/*U*_eq_	
O1	0.31502 (17)	1.03384 (16)	0.27815 (14)	0.0328 (3)	
O2	0.66529 (16)	0.87068 (16)	0.31015 (13)	0.0279 (3)	
C1	0.4547 (2)	0.8987 (2)	0.36257 (19)	0.0238 (3)	
C3	0.7222 (2)	1.0083 (2)	0.13405 (19)	0.0260 (3)	
H3A	0.6772	1.1460	0.1347	0.031*	
H3B	0.6496	1.0104	0.0423	0.031*	
C4	0.9628 (2)	0.9344 (2)	0.09306 (19)	0.0285 (4)	
H4A	1.0063	0.7942	0.0991	0.034*	
H4B	1.0334	0.9360	0.1842	0.034*	
C11	0.4134 (2)	0.7418 (2)	0.53811 (19)	0.0242 (3)	
C12	0.5775 (3)	0.6012 (2)	0.64157 (19)	0.0285 (3)	
H12	0.7208	0.6067	0.5976	0.034*	
N13	0.5455 (2)	0.4591 (2)	0.79911 (17)	0.0339 (3)	
C14	0.3422 (3)	0.4555 (2)	0.8578 (2)	0.0325 (4)	
H14	0.3158	0.3561	0.9703	0.039*	
C15	0.1685 (3)	0.5879 (2)	0.7646 (2)	0.0320 (4)	
H15	0.0269	0.5788	0.8116	0.038*	
C16	0.2046 (2)	0.7336 (2)	0.6017 (2)	0.0286 (4)	
H16	0.0883	0.8268	0.5342	0.034*	

### Atomic displacement parameters (Å^2^)

**Table:**

	*U*^11^	*U*^22^	*U*^33^	*U*^12^	*U*^13^	*U*^23^
O1	0.0257 (6)	0.0321 (6)	0.0288 (6)	−0.0071 (5)	−0.0041 (5)	−0.0009 (5)
O2	0.0222 (5)	0.0301 (6)	0.0229 (5)	−0.0088 (4)	−0.0001 (4)	−0.0023 (4)
C1	0.0231 (7)	0.0254 (7)	0.0224 (7)	−0.0086 (6)	−0.0017 (6)	−0.0075 (6)
C3	0.0264 (8)	0.0274 (7)	0.0194 (7)	−0.0117 (6)	0.0004 (6)	−0.0026 (6)
C4	0.0246 (8)	0.0301 (8)	0.0255 (8)	−0.0097 (6)	−0.0014 (6)	−0.0047 (7)
C11	0.0268 (8)	0.0216 (7)	0.0230 (7)	−0.0072 (6)	0.0006 (6)	−0.0090 (6)
C12	0.0260 (8)	0.0298 (8)	0.0248 (8)	−0.0075 (6)	0.0000 (6)	−0.0077 (6)
N13	0.0331 (7)	0.0303 (7)	0.0271 (7)	−0.0057 (6)	−0.0022 (6)	−0.0039 (6)
C14	0.0397 (9)	0.0266 (8)	0.0226 (8)	−0.0121 (7)	0.0039 (7)	−0.0032 (6)
C15	0.0293 (8)	0.0314 (8)	0.0308 (8)	−0.0135 (7)	0.0054 (6)	−0.0082 (7)
C16	0.0261 (8)	0.0264 (8)	0.0279 (8)	−0.0065 (6)	−0.0024 (6)	−0.0065 (6)

### Geometric parameters (Å, º)

**Table:**

O1—C1	1.2017 (18)	C11—C16	1.384 (2)
O2—C1	1.3342 (18)	C11—C12	1.389 (2)
O2—C3	1.4538 (17)	C12—N13	1.333 (2)
C1—C11	1.4886 (19)	C12—H12	0.9500
C3—C4	1.502 (2)	N13—C14	1.337 (2)
C3—H3A	0.9900	C14—C15	1.379 (2)
C3—H3B	0.9900	C14—H14	0.9500
C4—C4^i^	1.523 (3)	C15—C16	1.378 (2)
C4—H4A	0.9900	C15—H15	0.9500
C4—H4B	0.9900	C16—H16	0.9500
			
C1—O2—C3	115.98 (12)	C16—C11—C12	118.20 (14)
O1—C1—O2	124.15 (13)	C16—C11—C1	119.43 (13)
O1—C1—C11	123.99 (13)	C12—C11—C1	122.38 (13)
O2—C1—C11	111.87 (12)	N13—C12—C11	123.74 (14)
O2—C3—C4	106.96 (12)	N13—C12—H12	118.1
O2—C3—H3A	110.3	C11—C12—H12	118.1
C4—C3—H3A	110.3	C12—N13—C14	116.78 (14)
O2—C3—H3B	110.3	N13—C14—C15	123.82 (14)
C4—C3—H3B	110.3	N13—C14—H14	118.1
H3A—C3—H3B	108.6	C15—C14—H14	118.1
C3—C4—C4^i^	110.76 (16)	C16—C15—C14	118.61 (15)
C3—C4—H4A	109.5	C16—C15—H15	120.7
C4^i^—C4—H4A	109.5	C14—C15—H15	120.7
C3—C4—H4B	109.5	C15—C16—C11	118.86 (15)
C4^i^—C4—H4B	109.5	C15—C16—H16	120.6
H4A—C4—H4B	108.1	C11—C16—H16	120.6
			
C3—O2—C1—O1	−3.9 (2)	C16—C11—C12—N13	−0.3 (2)
C3—O2—C1—C11	175.89 (12)	C1—C11—C12—N13	−179.97 (15)
C1—O2—C3—C4	−173.02 (12)	C11—C12—N13—C14	−0.2 (2)
O2—C3—C4—C4^i^	177.89 (15)	C12—N13—C14—C15	0.5 (2)
O1—C1—C11—C16	7.7 (2)	N13—C14—C15—C16	−0.4 (3)
O2—C1—C11—C16	−172.10 (13)	C14—C15—C16—C11	−0.1 (2)
O1—C1—C11—C12	−172.59 (15)	C12—C11—C16—C15	0.4 (2)
O2—C1—C11—C12	7.61 (19)	C1—C11—C16—C15	−179.89 (14)

Symmetry code: (i) −*x*+2, −*y*+2, −*z*.
